# Porous Ceramic Sternal Prosthesis Implantation in a 13-Year-Old Patient Presenting with Metastatic Ewing's Sarcoma

**DOI:** 10.1055/s-0041-1740328

**Published:** 2022-01-15

**Authors:** Nicolas Mainard, Dyuti Sharma, Damien Fron, Aurélie Mezel, Federico Canavese, Michel Bonnevalle, Eric Nectoux

**Affiliations:** 1 Department of Pediatric Surgery, Jeanne de Flandre Hospital, Lille, France; 2 Université Lille 2 Droit et Santé Faculté de Médecine Henri Warembourg, Lille, Hauts-de-France, France; 3 Department of Pediatric Surgery, Reference Center for Congenital and Malformative Esophageal Disorders, Jeanne de Flandre Children's Hospital, Lille University Faculty of Medicine, Lille, France

**Keywords:** Ewing's sarcoma, sternum, prosthesis, bioceramics, tissue engineering

## Abstract

Ewing's sarcoma is the second most frequent primary malignant bone tumor in adolescents and young adults. Locations on the thoracic wall represent up to 20% of primary and secondary locations. We present the case of a 13-year-old patient treated with the use of a radiolucency porous bioceramic prosthesis as a sternal replacement for a wide tumor resection in an oncologic context. Focal radiation therapy was not possible due to the high risk of severe myocardial injuries caused by the sternal location of the tumor. The sternum CERAMIL® (I.CERAM, Limoges, France), in porous alumina (Al _2_ O _3_ ) has already been implanted into adults in sternal replacement during its invasion by a tumor or its infectious destruction. There were no complication concerning the surgery. The last follow-up at 2 years postoperatively reveals a satisfactory clinical situation with any functional thoracic complaint and nor any functional respiratory symptoms. The porous alumina sternal prosthesis offers a reliable alternative for sternal replacement indications for children in an oncologic context.

## Introduction

Ewing's sarcoma is the second most frequent primary malignant bone tumor in adolescents and young adults. 1 2 Between 80 and 100, new patients are reported each year in France. 3 It has a remotely strong invasive power, at the time of diagnosis ∼20 to 25% patients present with metastatic disease. Metastasis usually occurs to the lungs (70–80%) and to the bone (40–45%). 4 Locations on the thoracic wall represent up to 20% of primary and secondary locations. 5 The treatment is based on a general chemotherapy combined with a wide resection of the tumor which can lead to a massive loss of bone substance, thus a reconstruction. The development of biomaterial and the evolution of tissue engineering offer a promising solution to the replacement and transplantation of defective tissues within the human body. The objective is to produce devices with the potential to integrate and regenerate a specific functional tissue during their in vivo implantation.

Among these materials, bioceramics were established as an alternative to other polymers or metals for the replacement of bone structures. In 2005, Baino et al 6 stated that the main qualities of a biomaterial used in vivo are biocompatibility, optimal geometry allowing the guidance of tissue reconstruction, bioactivity stimulating the integration/incorporation of an implant, biomechanical qualities about the tissue it aims at replacing and biological properties allowing the development of angiogenesis and the stimulation of cell differentiation.

Here, we deal with the use of a radiolucency porous bioceramic prosthesis as a sternal replacement of a wide tumor resection in an oncologic context.

## Case Report

**Fig. 1:**
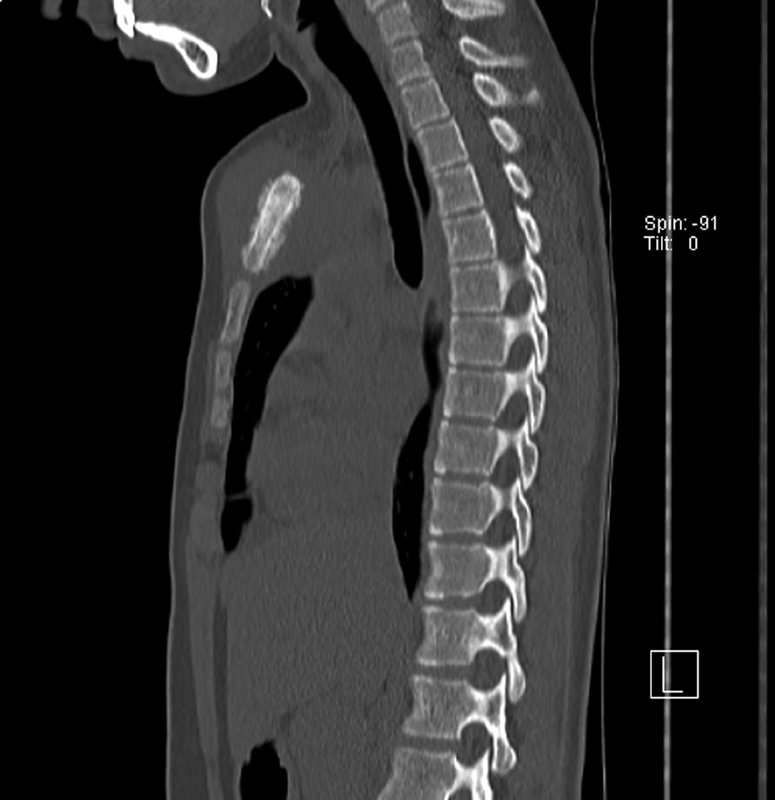
Preoperative computed tomography scan view of the sternum tumor.

In January 2019, our department of pediatric surgery provided care for a 13-year-old patient with metastatic Ewing's sarcoma and a primary lesion on the manubrium of sternum spread all over the mesosternum diagnosed 6 months earlier ( Fig. 1 ).

**Fig. 2:**
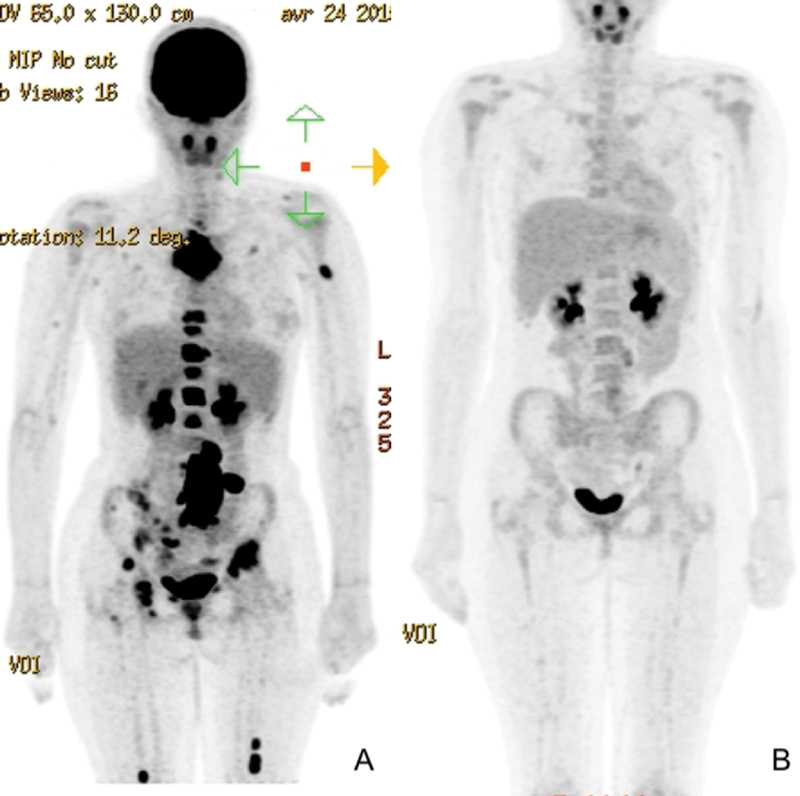
( **A** ) Positron emission tomography (PET) scan prechemotherapy. ( **B** ) PET scan postchemotherapy.

The patient presented with pulmonary metastasis and a lumbar intramuscular. She was treated with a neoadjuvant chemotherapy according to the COMBINAIRE R3 protocol in which she was included allowing the regression of most metastases except for a secondary ischial lesion ( Fig. 2 ).

**Fig. 3:**
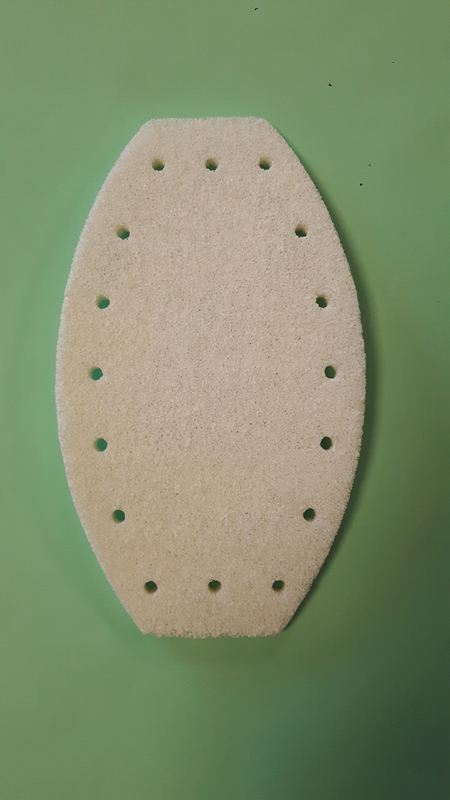
Ex vivo image of the implant.

Focal radiation therapy was not possible due to the high risk of severe myocardial injuries caused by the sternal location of the tumor. A wide sternum resection was done with a replacement by a porous ceramic prosthesis (sternum CERAMIL; I.CERAM, Limoges, France) ( Fig. 3 ).

**Fig. 4:**
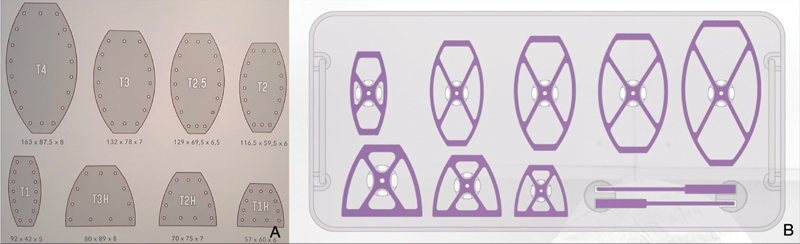
( **A** ) Available implant sizes. ( **B** ) Ancillary of trial implants.

The implant is available in five sizes and three half sizes for replacement of the manubrium. It is delivered with trial implants allowing the choice of the size and the optimization of preparation of the implantation area ( Fig. 4 ).

It is also possible to order custom implants for the patient if the clinical situation requires it.

Following the midline incision from the suprasternal notch to the xiphoid process, the pectoralis major muscle was sticking out on its whole sternal insertion revealing the costal cartilages from C1 to C8. A subperichondrial resection of the middle portion of all the costal cartilages as well as the two collarbones with clavicular osteotomy were performed. The sternum was raised like a hood.

**Fig. 5:**
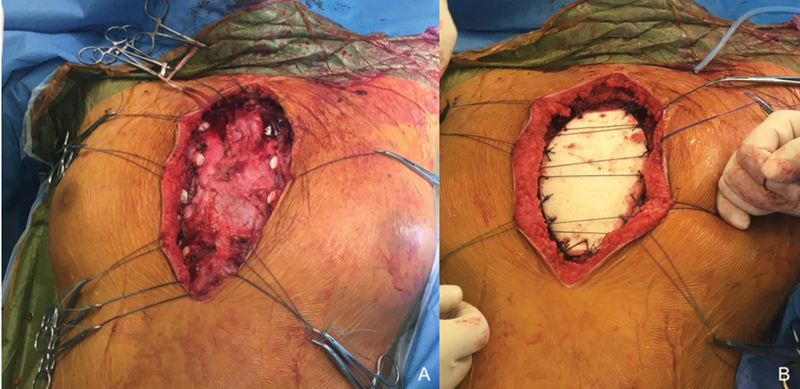
( **A** ) Intraoperative view after sternum resection. ( **B** ) Intraoperative view of I.Ceram porous alumina sternal prosthesis insertion with sutures.

The preperforated sternal prosthesis CERAMIL was positioned in place of the removed sternum and was successively attached to each costal cartilage stump with a nonabsorbable suture in no. 3 Mersuture type polyester. The sutures were loose in order, on the one hand, to preserve the mobility of the thorax and, on the other hand, to avoid the section of the wire on the ceramic matrix ( Fig. 5 ).

After its positioning, the prosthesis was relatively mobile compared with deep layers, but the overall showed a satisfactory rigidity. A chest Blake drain placed under the prosthesis at the end of the surgical procedure was removed on day 6. There were no complications in the early management of the patient.

**Fig. 6:**
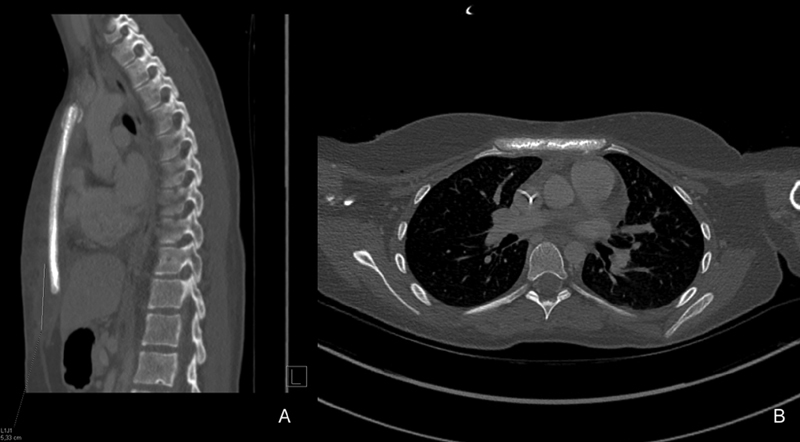
Three-week postoperative computed tomography scan.

At week 3, the evolution was satisfactory: a nocturnal noninvasive ventilation (NNIV) was still necessary, but the patient had no respiratory trouble. NNIV was maintained for 6 weeks. The local morphological appearance was satisfactory with admitted cutaneous healing. On palpation, the sternum was closely linked to the rib cage with no abnormal mobility. The patient did not complain of any pain. A computed tomography (CT) scan was performed 3 weeks postoperatively when mediastinitis was suspected. An implant in place was found without scannographic artifact allowing a precise study of the position of the implant and the surrounding structures ( Fig. 6 ).

The microscopic analysis of the sternal resection revealed a scar tissue and a necrotic appearance of the lesion without any residual viable tumor cell. The lesion was completely removed, with clear margins.

**Fig. 7:**
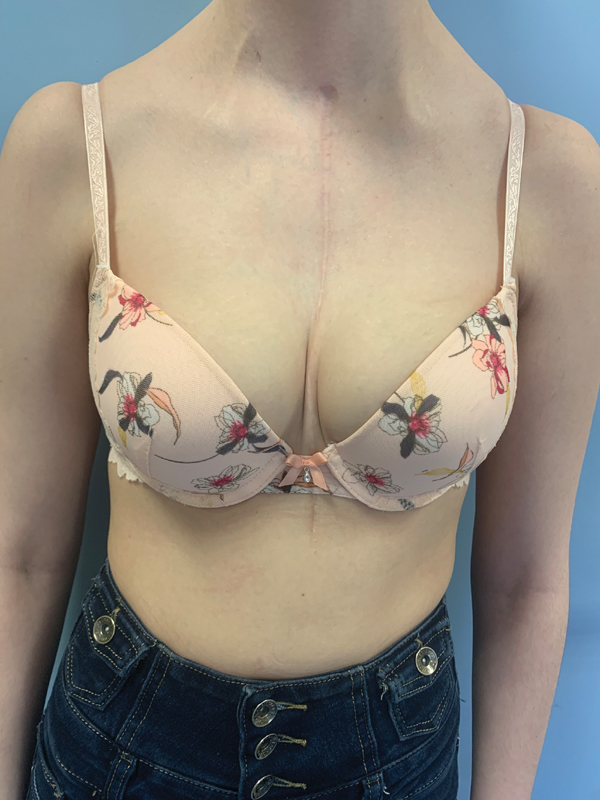
Appearance of the thorax at 28 months postoperatively.

At the 28-month follow-up, the patient did not present any functional thoracic complaint and nor any functional respiratory symptoms. There was no secondary displacement of the prosthesis, no chronic pericardial irritation or pleural effusion. The patient was satisfied with the clinical result and the morphological aspect of her thorax and could take part in leisure physical activities. Her clinical condition was compatible with good tolerance of this type of implant in children ( Fig. 7 ).

## Discussion

To this day, there is no referential approach for sternal reconstruction. Several techniques have been used such as bone graft, the use of metallic-structured implants or muscular flaps. 7 8 9 None is yet fully satisfactory to this indication. Indeed, the ideal characteristics for a sternal reconstruction prosthesis are a rigidity allowing the protection of mediastinal organs, a malleable inclination to maintain a satisfactory mechanical ventilation, radiolucency to create an anatomic reference to do a better follow-up and identify a possible local neoplastic relapse and a bioinertia to allow the in-growth of fibrous tissue and decrease the likelihood of infection. 10 11 The CERAMIL® sternum in porous alumina (Al _2_ O _3_ ) has already been implanted into adults in sternal replacement during its invasion by a tumor or its postoperative cardiac infectious destruction with a median follow-up of 18 months showing a satisfactory evolution. 12 No complication, especially infectious, was observed during this study.

Fouilloux et al 13 mentioned in 2019 the first implantation case in a 9-year-old child showing a congenital sternum agenesis with no postoperative complication of the implant after 12 months.

Denes et al, 14 as a commentary to the article of Baino et al, pointed out that this device meets the main qualities of a bioceramic. Its porous structure (a diameter ranging from 100 to 900 μm) allows a quick invasion of the ceramic by the osteoblasts allowing the biointegration on a long-term basis and as a result the stability of the ceramic within the bone while maintaining its biomechanical properties similar to the bone tissue (the mechanical resistance to compression being of 20 MPa). This balance between the porosity of the material and its mechanical properties is a crucial aspect for its bone application. Biocompatibility and bioinertia were established in the long term by the implantation of more than 5,000 bioceramic devices.

These metastatic pulmonary lesions represent 50% of secondary lesions in Ewing's sarcoma. 1 15 Such a prosthesis has the advantage of being radiolucent and not creating artifacts in case of a CT scan. In the context of postoperative oncology monitoring of pulmonary metastases from Ewing's sarcoma, this has a clear advantage over titanium prostheses.

It has been recently shown that bacterial adhesion is less important on porous alumina than on other materials such as titanium or stainless steel, 16 17 which is another asset in surgeries with risks of infection such as wide oncologic excision of the sternum.

## Conclusion

The alumina sternal prosthesis appears to be a reliable option in sternal replacement indications for children. This first case of implantation of a porous alumina prosthesis in an oncologic context confirms it. However, a long-term follow-up for this implant seems must be required.

## Notes

New Insights & the Importance for the Pediatric Surgeon We present the use of a radiolucency porous bioceramic prosthesis in pediatrics as a sternal replacement for a wide tumor resection in an oncologic context. This is the first use in this indication. Outcomes are excellent.
